# Determination of Total Selenium in Infant Formulas: Comparison of the Performance of FIA and MCFA Flow Systems

**DOI:** 10.1155/2012/918292

**Published:** 2012-02-20

**Authors:** Mariela Pistón, Moisés Knochen

**Affiliations:** Department of Analytical Chemistry, School of Chemistry, Universidad de la República (UdelaR), Avenue Gral. Flores 2124, 11800 Montevideo, Uruguay

## Abstract

Two flow methods, based, respectively, on flow-injection analysis (FIA) and on multicommutated flow analysis (MCFA), were compared with regard to their use for the determination of total selenium in infant formulas by hydride-generation atomic absorption spectrometry. The method based on multicommutation provided lower detection and quantification limits (0.08 and 0.27 **μ**g L^−1^ compared to 0.59 and 1.95 **μ** L^−1^, resp.), higher sampling frequency (160 *versus*. 70 samples per hour), and reduced reagent consumption. Linearity, precision, and accuracy were similar for the two methods compared. It was concluded that, while both methods proved to be appropriate for the purpose, the MCFA-based method exhibited a better performance.

## 1. Introduction

The importance of selenium for the life of animals has been known since the 1950s, while the essentiality of this element for humans was revealed in 1970s with the discovery of its ability to prevent the Keshan disease, first described in 1935 in China in a province of the same name [1].

Subsequently, it was discovered its important role as part of the body's antioxidant mechanisms and also its role in protecting the body against heavy metals as well as its importance in the immune system [1, 2].

Cow's milk and dairy products are usually a poor source of selenium in human nutrition. Human milk is considered the best source of selenium for young children and infants [3]. However, human milk is a poor source of selenium, so recently manufacturers of dairy-based infant formulas have added selenium to these products.

Selenium has a very narrow range between dietary deficiency (<40 *μ*g/day for adults) and toxic levels (>400 *μ*g/day). For this reason, it is very important to have control of the selenium intake by human and animals.

Several analytical techniques are suitable for determining total selenium in milk, such as neutron activation analysis (Instrumental Neutron Activation Analysis (INAA)), fluorimetry, gas chromatography (GC), and atomic absorption spectrometry (AAS) [4].

The neutron activation technique is not available in most laboratories; it is generally used for determinations of selenium in certified reference materials as an alternative method and in proficiency testing. It has the advantage of being a nondestructive technique, but the detection limits are usually higher than for other techniques [3, 4].

Other methods usually are based on the generation of a piazselenol complex, which is subsequently submitted to different detection techniques.

For instance, a fluorimetric method is recommended in the bulletin of the International Dairy Federation (IDF) [5] and in the AOAC 1996 compendium as an official method [6], thus it has been widely studied. The method is based on the measurement of fluorescence of a piazselenol formed from selenite and DAN (2,3-diaminonapthalene) which is a carcinogenic reagent. Detection limits as low as 0.2 *μ*g L^−1^ can be obtained; this is suitable for this kind of determinations but it involves a stage of prereduction and extraction of the complex in an organic solvent [3, 4].

Another method for selenium determination is based on the reaction with (3,3′-diaminobenzidine) in acidic medium to form the corresponding piazselenol, which is measured spectrophotometrically. In this method, the necessary time for color development is 50 minutes [7].

As an example of determination of selenium by gas chromatography, we can mention the method based on selective complexation of selenium to form the piazselenol. This is extracted into an organic phase before being injected in a chromatograph fitted with an electron capture detector [4].

The determination can be performed also by HPLC using fluorescence detection [4]. Chromatographic methods are an interesting alternative to study speciation of selenium in milk.

The determination of total selenium in milk at trace and ultratrace levels is often performed by electrothermal atomic absorption spectrometry (ET-AAS) [8, 9]. Other popular technique is atomic absorption spectrometry with hydride generation (HG-AAS) [10–12]. This technique also presents some important advantages, including the separation of the analyte from the matrix which reduces the number of interferences that may occur.

The generation of the hydride can be carried out in batch or can be automated using different flow systems.

Continuous flow systems have been used extensively for hydride generation for over 20 years, and there are several commercial models with accessories for hydride generation [13–15].

Automation by flow injection analysis (FIA) technique [16, 17] has proved to be useful and efficient for this purpose; several articles published report the coupling of FIA to the generation of hydrides with excellent results (FIA-HG-AAS) [12, 18].

Both continuous flow and FIA systems have also been coupled to atomic florescence detection (FIA-AFS) [19–21].

There are also some reports of flow systems coupled with atomic emission spectrometry (HG-ICP-OES) [22, 23].

Semenova et al. reported an application for total inorganic selenium determination by hydride generation-atomic fluorescence spectrometry with a multisyringe flow injection system (MSFIA) [24].

On the other hand, multicommutated flow analysis (MCFA) is a technique based on flow networks built around electrically operated solenoid valves which are turned on or off under computer control [25–28]. It has been already used by Ródenas-Torralba et al. for the determination of tellurium in milk by hydride generation but coupled to atomic fluorescence detection [21]. In a previous work we developed and validated a multicommutated flow system for the determination of total selenium in milk and infant formulas [29].

When comparing the techniques of FIA and MCFA, it is evident that FIA systems tend to be simpler and can be implemented with a manually operated valve, although such a system is not useful if some degree of automation is desired. For this purpose, however, an electrically operated valve can be used connected to a computer or even to an appropriate timer with an electric switch. MCFA on the other hand requires some electronic and computer skills but the user ends up with a much more flexible system which is intrinsically amenable to automation. Given that the solenoid valves are controlled by the software, it is easy to modify the time when each valve is energized and the duration of that condition. This enables to change sample and reagent volumes, reaction times, and also, by resorting to binary sampling, the form in which samples and reagents are mixed.

The aim of this work is the comparison and evaluation of the performance of two flow systems based on different techniques: a FIA system developed, optimized, and validated, presented in this work and a MCFA system developed and validated in a previous work [29]. This comparison is proposed in order to investigate the benefits that either technique could provide for the determination of total selenium in infant formulas. The advantages and disadvantages of each in terms of figures of merit are discussed.

The two flow systems were operated using detection by atomic absorption with hydride generation, allowing the determination of selenium as Se(IV) which is the species that can be detected as SeH_2_. The Se(IV) was obtained by quantitative reduction of total inorganic selenium present in the matrix after mineralization.

Interferences due to the presence of transition metals were not expected due to the low concentrations of these potential interferents in this matrix; a fact that has been confirmed in the literature [30–32].

## 2. Experimental

All glassware was soaked overnight in 10% (v/v) nitric acid and then rinsed exhaustively with deionized water.

Connections and doubly-helical mixing coils were made from 0.8 mm internal diameter Teflon PFA tubing.

A U-shaped lab-built glass gravitational gas-liquid phase separator was used. The carrier gas was nitrogen (dried and purified by a combined Drierite/molecular-sieve trap).

### 2.1. Reagents

Sodium tetrahydroborate (hydride-generation grade) was obtained from Fluka. A 0.5% (w/v) solution was prepared daily by dissolving the solid in 0.05% (w/v) NaOH. All other reagents were of analytical reagent grade.

Purified water (ASTM Type I) was obtained from a Millipore (São Paulo, Brazil) Simplicity 185 purifier fed with glass-distilled water. A 1000 mg L^−1^ selenium standard solution was prepared from selenium metal (Aldrich, 99.99%), dissolved in nitric acid, and made up to volume with 10% (v/v) hydrochloric acid. An intermediate standard solution (0.8 mg L^−1^) was prepared daily by stepwise dilution with 1.5% (v/v) hydrochloric acid. Calibration solutions were prepared by dilution of the intermediate solution.

Measurements were carried out with a Perkin Elmer (Norwalk, CT, USA) model 5000 atomic absorption spectrometer fitted with a 10 cm burner (air-acetylene flame) and operated at the 196.0 nm analytical line. Atomization was carried out in a T-shaped quartz atomization cell (Precision Glassblowing, Centennial, CO, USA). The light source was a Photron (Narre Warren, Australia) Superlamp intensified emission hollow-cathode lamp operated as recommended by the manufacturer.

### 2.2. Calibration

Calibration solutions were prepared by accurately diluting aliquots of the 0.8 mg L^−1^ intermediate standard solution, to which 20 mL of water and 10 mL of concentrated hydrochloric acid were added. The mixture was heated on a hot plate for 1 hour at gentle boiling to carry out the prereduction of Se(VI) to Se(IV) then cooled down to room temperature and diluted to 30.0 mL with purified water.

### 2.3. Sample Preparation

The sample used for this comparative study was a standard reference material (SRM) of infant formula (NIST 1846 Infant Formula). These were prepared as follows: 0.50 g of the sample was accurately weighed in a 30 mL screw-capped Teflon PFA vessel (Savillex, Minnetonka, MN, USA). Then, 6 mL of concentrated nitric acid was added, the vessel was loosely capped, placed in a modified polypropylene “fast cooker”, and heated in a household microwave oven (Ariston model MO991B). The cooker was modified in order to vent all acid vapors and other gases via a piece of tubing to a flask containing sodium hydroxide solution which acted as a trap for acidic vapors. The oven was programmed to heat for 5 minutes at 30% and then for 3 minutes at 40% of the maximum power. It was then cooled down to room temperature, 1 mL of 30% hydrogen peroxide was added, and the vial (loosely capped) was heated again for 2 minutes at 40% power. Afterwards, the contents of the vial were transferred quantitatively to a 50 mL Erlenmeyer flask containing 10 mL of 10% (w/v) sulfamic acid solution and 10 mL of concentrated HCl; the prereduction step was carried out by heating at gentle boiling on a hot plate for 1 hour and then cooled down to room temperature and diluted with water to 20.0 mL [29].

### 2.4. Flow Systems

**Figure 1:**
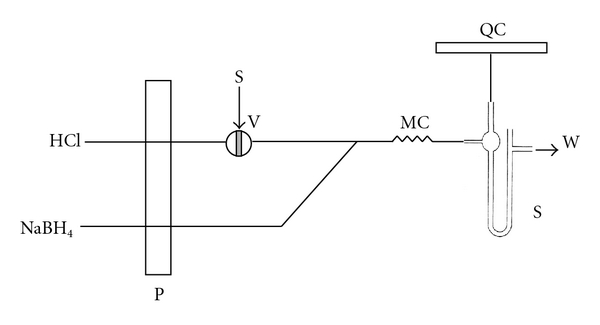
FIA system for total selenium determination by HG-AAS. S: sample; V: 6-port valve; P: peristaltic pump; MC: mixing coil (50 cm); PS: phase separator; QC: quartz cell; W: waste.

The FIA system (Figure 1) was based upon a Gilson (Villiers-le-Bel, France) Minipuls 2 multichannel peristaltic pump fitted with either Tygon or Viton tubing. Injection of the sample was made by means of a 6-port Valco Cheminert valve with microelectric actuator controlled from a personal computer via the serial RS232 serial port using a program compiled in QuickBasic 4.0.

**Figure 2:**
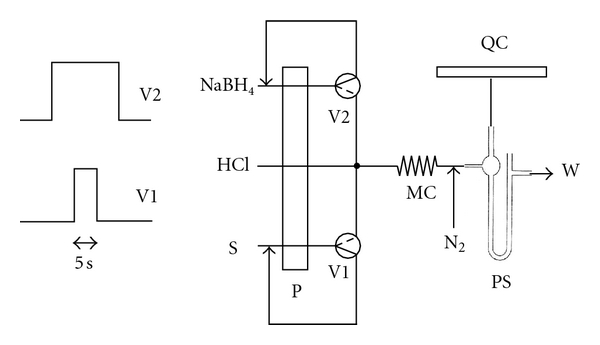
Multicommutated flow system. On the left the time sequence. S: sample (7.2 mL min^−1^), MC: mixing coil (50 cm), PS: phase separator, QC: quartz cell, W: waste, and P: peristaltic pump. V1, V2: solenoid valves. HCl: 5% (v/v): 3.2 mL min^−1^; NaBH_4_: 0.5% (w/v) solution in 0.05% (w/v): 1.7 mL min^−1^. N_2_: carrier gas (nitrogen), 0.32 L min min^−1^.

The MCFA system (Figure 2) was based on a peristaltic pump and two 3-way solenoid valves; it has been already described elsewhere [29].

For both systems, the analytical signal (absorbance) was obtained from the analog output connector of the spectrometer (1-V full scale) and digitized via a 12-bit analog to digital interface (Measurement Computing, model USB 1208LS) connected to a USB port and operated at a sampling rate of 1 s^−1^. A program was compiled in Visual Basic 6.0 for this purpose.

#### 2.4.1. FIA System

Figure 1 shows a schematic design of the FIA system. In this system, the sample is introduced into the flow stream of a carrier using a two-position injection valve. The loading time of the loop and subsequent injection of the sample by changing the position of the valve automatically were programmed in the software. Once the analytical signal returns to the baseline and the recording finishes, a new cycle of injection starts.

For optimization, two multivariate experiments (based on a central composite design) [34] and several univariate experiments were carried out.

In Table 1, the influence of the flow rate of the carrier (HCl), the reducing agent (NaBH_4_), and the mixing coil length in the peak height (signal) are presented. For these experiments a solution of Se(IV) of 50 *μ*g L^−1^ was prepared. The fixed variables were the concentrations of the carrier and the reducing agent (HCl: 10% (v/v); NaBH_4_: 0.2% (m/v)), the sample volume (500 *μ*L), and the flow rate of the carrier gas (N_2_: 0.20 L min^−1^).

According to the results in Table 1, experiment 3 determined that the best conditions were mixing coil length 50 cm, carrier flow rate 3.5 mL min^−1^, and flow rate of the reducing agent 4.5 mL min^−1^.

To complete the final optimization, a central composite design [34] for 4 variables and 3 levels was planned.

Table 2 shows the 3 levels of the variables to consider. In this experiment the fixed conditions were mixing coil length (50 cm), carrier gas flow rate (N_2_: 0.20 L min^−1^), and sample volume (500 *μ*L).

Table 3 shows the results of the 17 experiments of the proposed design. Experiment number 11 proved to give the best results; this meant a significant decrease in the concentration of HCl and NaBH_4_ with respect to the initial conditions.

Once the optimal operative conditions were reached, the sampling frequency was 70 hours^−1^.

#### 2.4.2. Multicommutated System (MCFA)

A rigorous description of the optimization of this system is presented in a previous work [29] using multivariate experiments. Figure 2 shows the scheme of the MCFA system.

Under the conditions shown in Figure 2, the sampling frequency was 160 samples per hour (hour^−1^).

## 3. Validation

Linearity was studied by means of an 8-point calibration curve in the range of 1.0–50.0 *μ*g L^−1^ (for both systems: *n* = 5). The linearity range was evaluated by visual inspection of the graphical representation and by means of the regression coefficient.

The routine calibration curve covered a smaller range because selenium concentrations expected following the preparation of these samples did not exceed 5 *μ*g L^−1^. For this reason a routine calibration curve in the range of 1–10 *μ*g L^−1^ was adequate.

Precision (*s*_*r*_  (%)) was estimated by analytical repetition of the complete analysis of the reference material (*n* = 5).

Detection (LD, 3*σ*) and quantification (LQ, 10*σ*) limits were estimated by measuring (*n* = 10) the dispersion of the blank and signal referring the measurements to the calibration curve.

The figures of merit are presented in Table 4.

To establish the trueness of the proposed methods, a certified reference material of infant formula (NIST 1846 Infant Formula) was analyzed.

Trueness was evaluated by comparison of the value of total selenium obtained for each system (*n* = 5) with the reference value of the SRM by means of a Student's *t*-test (Table 5) [33].

No evidence was found of the existence of significant interference in this matrix. This can be justified because of the low concentrations of potential interferents (transition metals), in this kind of matrix. Results are presented in Table 5.

## 4. Results and Discussion

Table 4 shows the figures of merit obtained from the validation of the FIA and MCFA systems for the determination of total selenium in reference material of infant formula.

The results in Table 5 show that values obtained using both systems do not present bias, thus the trueness was demonstrated.

The most impressive figures of merit were the limits of detection and quantification in solutions for MCFA-HG-AAS, which were almost one order of magnitude lower than the values for FIA-HG-AAS, and the sampling frequency that was more than twice using the MCFA system. The sampling rate was even higher than the reported by Semenova et al. for a MSFIA system.

This added to the lower reagent and sample consumption (and hence waste generation) of the MCFA system which is in accordance with the principles of Green Chemistry.

In terms of precision and accuracy, both techniques are appropriate for the proposed application.

The FIA method exhibited a greater linear range, but the difference is not significant for this application.

Commercial continuous flow systems are widely used, but they consume a large amount of sample and reagents since they are continuously circulating through the system while the determination is carried out. In addition, a single determination generally takes more than a minute.

The rigorous assessment of the figures of merit for validation after the optimization of a flow system for a particular application is important to develop new fast, reliable, and environmentally friendly analytical methods.

## 5. Conclusions

The two flow systems developed were successful for the determination of total selenium in infant formulas by hydride generation atomic absorption spectrometry.

The multicommutated flow system (MCFA) showed advantages over the flow injection system (FIA), presenting a much better detection limit and a higher analytical throughput. It also generated less chemical waste thus being more environmentally friendly.

The MCFA system also demonstrated to be more flexible because of the possibility to easily change operating parameters such as sample volume by means of the software user interface, without the need of physical modifications of the flow system.

## Figures and Tables

**Table 1:** Influence of the FIA system variables: multivariate design.

Experiment	Flow rate HCl (mL min^−1^)	Flow rate NaBH_4_ (mL min^−1^)	Mixing coil length (cm)	Absorbance Se(IV) 50 *μ*g · L^−1^ dissolution (*n* = 3)
1	1.3	1.7	150	0.044
2	2.9	3.6	150	0.069
**3**	**3.5**	**4.5**	**50**	**0.076**
4	1.3	1.7	100	0.047
5	2.9	3.6	100	0.069
6	2.2	2.8	100	0.062
7	3.5	4.5	100	0.072

**Table 2:** Variables and levels for the experimental design.

Variable	Level 1	Level 2	Level 3
Flow rate HCl (mL min^−1^)	1.3	2.8	5.9
Flow rate NaBH_4_ (mL min^−1^)	1.7	3.7	4.5
HCl% (v/v)	5	15	30
NaBH_4 _(% (m/v))	0.1	0.2	0.5

**Table 3:** Central composite experimental design, 4 variables and 3 levels.

Experiment	Flow rate HCl (mL min^−1^)	Flow rate NaBH_4_ (mL min^−1^)	HCl% (v/v)	NaBH_4_ (% (m/v)) in NaOH 0.05%	Absorbance Se(IV) 50 *μ*g · L^−1^ (*n* = 3) dissolution
1	1.3	1.7	5	0.1	0.076
2	1.3	1.7	5	0.5	0.069
3	1.3	1.7	30	0.1	0.081
4	1.3	1.7	30	0.5	0.070
5	1.3	4.5	5	0.1	0.065
6	1.3	4.5	5	0.5	0.043
7	1.3	4.5	30	0.1	0.066
8	1.3	4.5	30	0.5	0.044
9	2.8	3.7	15	0.2	0.167
10	5.9	1.7	5	0.1	0.222
**11**	**5.9**	**1.7**	**5**	**0.5**	**0.228**
12	5.9	1.7	30	0.1	0.205
13	5.9	1.7	30	0.5	0.200
14	5.9	4.5	5	0.1	0.162
15	5.9	4.5	5	0.5	0.112
16	5.9	4.5	30	0.1	0.170
17	5.9	4.5	30	0.5	0.115

**Table 4:** Figures of merit: comparison of performance of the FIA and MCFA flow systems.

Parameter	FIA system	MCFA system
Detection limit (LD) (*3*σ*/slope of calibration, * *n* = 10)	0.59 *μ*g L^−1^ in solution	0.08 *μ*g L^−1^ in solution
Quantification limit (LQ) (*10*σ*/slope of calibration, * *n* = 10)	1.95 *μ*g L^−1^ in solution	0.27 *μ*g L^−1^ in solution
Linearity (*μ*g · L^−1^)	2–50 (*r*_2_ = 0.999)	0.27–27 (*r*_2_ = 0.999)
Precision *s*_*r*_ (%) (*n* = 5)	<10	<10
Sampling frequency (hour^−1^)	70	160
Sample consumption per determination (mL)	0.5	0.6
Reagent consumption for each determination	0.25 mL HCl* NaBH_4_: 7.3 mg *concentrated	0.05 mL HCl* NaBH_4_: 2.5 mg *concentrated

**Table 5:** Total selenium contents found in standard reference material for both flow systems. Total selenium contents found in standard reference material and comparison with reference value by Student's *t*-test. s: standard deviation. *t*(0.05,4) = 2.78 [33].

Certified reference material	Certified value (mg kg^−1^)	MCFA found (mean ± s) (mg kg^−1^) (*n* = 5)	FIA found (mean ± s) ) (mg kg^−1^) (*n* = 5)	*t*-experimental
NIST 1846 (infant formula)	0.08 (*)	0.0807 ± 0.0075	0.0823 ± 0.0065	0.81 (FIA) 0.21 (MCFA)

(*) NIST information value. All the results are expressed in dry basis.
